# Evidence of neofunctionalization after the duplication of the highly conserved Polycomb group gene *Caf1-55* in the obscura group of *Drosophila*

**DOI:** 10.1038/srep40536

**Published:** 2017-01-17

**Authors:** Juan M. Calvo-Martín, Montserrat Papaceit, Carmen Segarra

**Affiliations:** 1 Departament de Genètica, Microbiologia i Estadística, Facultat de Biologia, i Institut de Recerca de la Biodiversitat (IRBio), Universitat de Barcelona, Barcelona, Spain

## Abstract

*Drosophila* CAF1-55 protein is a subunit of the Polycomb repressive complex PRC2 and other protein complexes. It is a multifunctional and evolutionarily conserved protein that participates in nucleosome assembly and remodelling, as well as in the epigenetic regulation of a large set of target genes. Here, we describe and analyze the duplication of *Caf1-55* in the obscura group of *Drosophila*. Paralogs exhibited a strong asymmetry in evolutionary rates, which suggests that they have evolved according to a neofunctionalization process. During this process, the ancestral copy has been kept under steady purifying selection to retain the ancestral function and the derived copy (*Caf1-55dup*) that originated via a DNA-mediated duplication event ~18 Mya, has been under clear episodic selection. Different maximum likelihood approaches confirmed the action of positive selection, in contrast to relaxed selection, on *Caf1-55dup* after the duplication. This adaptive process has also taken place more recently during the divergence of *D. subobscura* and *D. guanche*. The possible association of this duplication with a previously detected acceleration in the evolutionary rate of three CAF1-55 partners in PRC2 complexes is discussed. Finally, the timing and functional consequences of the *Caf1-55* duplication is compared to other duplications of Polycomb genes.

*Drosophila* CAF1-55 protein (also known as CAF1, p55 and NURF55) is part of the Polycomb repressive complex PRC2^1^,^2^. This protein complex is responsible for the trimethylation of the histone H3 lysine 27 (H3K27me3) that is one of the posttranslational histone modifications introduced by the Polycomb repressive complexes at specific target sites to modulate the chromatin state. However, CAF1-55 is a multifunctional protein and is a subunit of other protein complexes such as CAF1, NURF, NuRD and REAM/MMB. The heterotrimeric complex CAF1 (chromatin assembly factor 1) is involved in the assembly of nucleosomes after DNA replication^3^. NURF (nucleosome remodelling factor) is an ATP-dependent chromatin remodelling complex^4^ and NuRD is a nucleosome remodelling and deacetylase complex^5^. REAM (Rb, E2F and Myb complex) and MMB (Myb-MuvB complex) are two similar protein complexes that have been independently purified and participate in the activation and repression of developmental genes and origins of DNA replication^6^,^7^.

A common function of CAF1-55 in all these complexes is to serve as a scaffold to facilitate the interaction between histones and other proteins. Indeed, the interaction of CAF1-55 with the first helix of histone H4 and other proteins has been resolved by crystal structure analysis^8^. Structural analysis indicates that CAF1-55 is a noncatalytic protein and a member of the WD40 family with a seven-bladed β-propeller structure. WD40 proteins participate in protein–protein interactions, and they are overrepresented among proteins involved in interactome networks^9^. CAF1-55 interacts by means of the WD40 repeats with 35 different *D. melanogaster* proteins, as reported in the BioGRID database^10^.

The pivotal role of CAF1-55 in chromatin metabolism, as well as its ability to interact with a wide range of proteins, indicates that CAF1-55 is a hub protein with multiple pleiotropic effects, which makes it an essential protein. Indeed, *Caf1-55* null alleles cause lethality before pupariation and mutant larvae die mostly at the second instar stage^11^. Reduced levels of *Caf1-55* expression result in homeotic transformations likely due to missregulation of the Hox genes by PRC2^12^. Proteins with a large number of interactors, especially if they are located at the center of a network, are subject to strong constraints on variation and are evolutionarily conserved^13^, which is consistent with the presence of CAF1-55 in a wide range of species, from fungi to mammals and plants, and with its high level of amino acid conservation. In fact, the sequence identity between the *Drosophila melanogaster* CAF1-55 and the human homologs RbAp48 and RbAp46 is 87% and 86%, respectively^3^.

In a study of the molecular evolution of *Caf1-55* and other Polycomb group (PcG) genes in *Drosophila*^14^, the orthologs of *Caf1-55* were identified in a set of 15 species, and it was confirmed that CAF1-55 is a highly conserved protein with minimal interspecific amino acid divergence. In the same study, a gene with a high similarity with *Caf1-55* was unexpectedly detected in *D. pseudoobscura* and *D. persimilis*. This gene (henceforth, *Caf1-55dup*) was absent in the other *Drosophila* species sequenced by the *Drosophila* 12 Genomes Consortium^15^. This result suggested the duplication of *Caf1-55* prior to the divergence of the obscura group species. Herein, we analyze the molecular evolution of *Caf1-55* and *Caf1-55dup* paralogs both at the interspecific level in the *Drosophila* genus and at the intraspecific level in *D. subobscura* in order to infer the evolutionary history of this duplication. Different alternative scenarios can drive the evolution of duplicated genes: conservation (both copies retain the ancestral function in a redundant manner), nonfunctionalization (one copy retains the ancestral function and the other becomes silenced and degenerates), neofunctionalization (one copy retains the ancestral function and the other acquires a new function), subfunctionalization (both copies retain a different subset of the ancestral functions) and specialization (both copies acquire a novel function different from the ancestral one). Among these scenarios, only neofunctionalization and specialization permit the origin of new genes and functions^16^. Thus, the first aim of this study was to disentangle under which of these scenarios did *Caf1-55* and *Caf1-55dup* evolve. In fact, purifying selection acting on the ancestral gene may be relaxed after the duplication due to gene redundancy. This relaxation might have reduced the extent of conservation of CAF1-55. Moreover, this duplication might have increased the dosage of CAF1-55, which might have been a challenge for a protein involved in different complexes if it led to an imbalance in the concentration of the different subunits that form these complexes^17^.

The results obtained indicate that the duplication of *Caf1-55* occurred about 18 Mya in the lineage ancestral to the obscura group species and that both paralogs likely evolved under a neofunctionalization process, in which strong purifying selection was maintained on the ancestral *Caf1-55* gene and positive selection acted on the new *Caf1-55dup* gene. The action of positive selection on *Caf1-55dup* was not only detected immediately after its origin, but also more recently, specifically since the divergence of *D. subobscura* and *D. guanche*. In addition, the results show that *Caf1-55* is a dynamic gene, as it underwent at least an additional duplication event in the *D. persimilis* lineage (about 0.35 Mya).

## Results

### Identification of the *Caf1-55* orthologs and paralogs

*Caf1-55* orthologs in the species sequenced by the *Drosophila* 12 Genomes Consortium^15^ are described in FlyBase (www.flybase.org). However, BLASTN searches using as query the coding region of the *Caf1-55* gene of *D. melanogaster* (CG4236) were performed to corroborate the available data. The sequences with the highest similarity to the query and with E values close to 0 were GA18051 in *D. pseudoobscura* and GL12530 in *D. persimilis*. Synteny with flanking genes is conserved when comparing CG4236, GA18051 and GL12530, which confirmed that the three genes are orthologs. Unexpectedly, these BLASTN searches retrieved other genes with a rather high similarity to *Caf1-55* in *D. pseudoobscura* (GA26389; E value = 6.48 e^−12^) and *D. persimilis* (GL21757; E value = 2.47 e^−8^). These two genes are located in a conserved syntenic region that is different from that of *Caf1-55*. According to FlyBase, GA26389 or GL21757 have not orthologs in the other species sequenced by the *Drosophila* 12 Genomes Consortium. Additional BLASTN searches using as query these genes and analysis of the syntenic region where they are located corroborated that GA26389 or GL21757 are absent in these species. This result suggested a duplication of *Caf1-55* in the obscura group species, which was further confirmed by the presence of the *Caf1-55* duplicate, *Caf1-55dup*, in the available genome of *D. miranda*^18^ and by the successful PCR amplification and sequencing of *Caf1-55dup* in three additional species of the obscura group (*D. subobscura, D. madeirensis* and *D. guanche*). It is remarkable that *Caf1-55* has an additional duplicate (GL12106) in the annotated *D. persimilis* genome, which is misidentified as a *Caf1-55* ortholog in FlyBase. The presence of this paralog was confirmed by its PCR amplification and sequencing in a *D. persimilis* line available in our laboratory.

In *D. pseudoobscura, D. persimilis* and *D. miranda Caf1-55* and *Caf1-55dup* are located on chromosome 2 (Muller’s element E), about 8 Mb apart. *In situ* hybridization confirmed the location of both genes in the same chromosomal element (chromosome O) of *D. subobscura* (see Supplementary Fig. S1), in a region with a recombination rate of about 5 cM/Mb^19^. *Caf1-55dup* has three exons and two introns in the six obscura species and thus has kept the same organization of the ancestral gene, indicating a DNA-mediated duplication event. In *D. persimilis*, the annotated *Caf1-55dup* gene (GL21757) lacks the first exon. However, sequence homology indicates that this exon is present in the genomic sequence upstream from the gene, which clearly suggests a missannotation. In *D. pseudoobscura* and *D. persimilis, Caf1-55dup* is a nested gene inserted in the fourth intron of GA27362 and GL22062, respectively, these being orthologs of the *D. melanogaster* gene *dpr11* (CG33202). Moreover, the multiple alignment of the sequenced gene regions of *Caf1-55dup* in *D. subobscura, D. madeirensis* and *D. guanche* with the sequence of the fourth intron of *dpr11* in *D. melanogaster* shows regions with a clear homology flanking *Caf1-55dup*. Therefore, the genomic location of *Caf1-55dup* is maintained in the obscura group species, which indicates that this is its ancestral position at least prior to the divergence of these species.

The expression analysis (not including *D. miranda*) confirmed that *Caf1-55dup* is transcribed in the adult stage of the obscura group species (see Supplementary Fig. S2). Moreover, the *Caf1-55dup* cDNA sequences that were obtained lacked introns, supporting the notion that the recovered cDNA was from processed and likely functional mRNA.

### Divergence of *Caf1-55* and its paralogs

**Figure 1:**
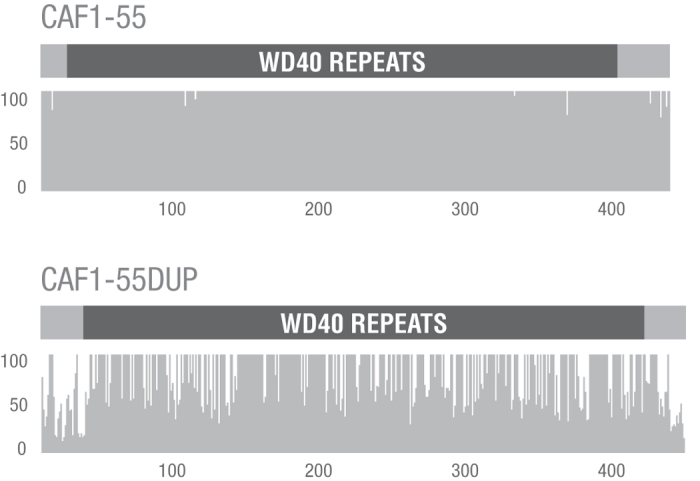
Protein conservation plots across CAF1-55 and CAF1-55DUP according to interspecific divergence. *x*-axis, amino acid sites along the multiple alignment of each protein. *y*-axis, inferred amino acid conservation score at each site. The grey bar above each plot shows the described WD40 domain in a black box.

The *Caf1-55* and *Caf1-55dup* sequences retrieved from FlyBase were aligned with those reported in Calvo-Martín *et al*.^14^ and in the present study. This alignment was based on a multiple alignment of the encoded proteins (see Supplementary Fig. S3), which included 23 sequences (16 CAF1-55, 6 CAF1-55DUP and the protein encoded by GL12106 that is the additional annotated *D. persimilis* duplicate of *Caf1-55*). Amino acid conservation is much higher along CAF1-55 than along CAF1-55DUP (Fig. 1) suggesting that purifying selection is stronger in *Caf1-55* than in its paralog. *Caf1-55dup* lacks the start codon in *D. miranda*, which was further confirmed by sequencing this gene in a line of this species available in our laboratory. Therefore, in *D. miranda Caf1-55dup* might be undergoing a process of pseudogenization or, alternatively, a different ATG may be in use as the start codon to translate the gene. In fact, an ATG in the first intron in frame with the second exon could render a CAF1-55DUP protein that only differs in the first five amino acids relative to that of *D. pseudoobscura* and *D. persimilis*. However, given this ambiguity and the possibility that *Caf1-55dup* is a pseudogene in *D. miranda*, this species was excluded from all subsequent analyses. On the other hand, the alignment of the *Caf1-55* and *Caf1-55dup* 5′ flanking regions in the obscura group species revealed that sequence homology extends only to around the transcription start site and thus that the duplicated region includes the *Caf1-55* 5′ UTR but not the upstream sequences. The absence of the promoter and other regulatory sequences in the duplicated region suggests that current expression of *Caf1-55dup* is directed by newly arisen *cis*-acting regulatory elements.

**Figure 2:**
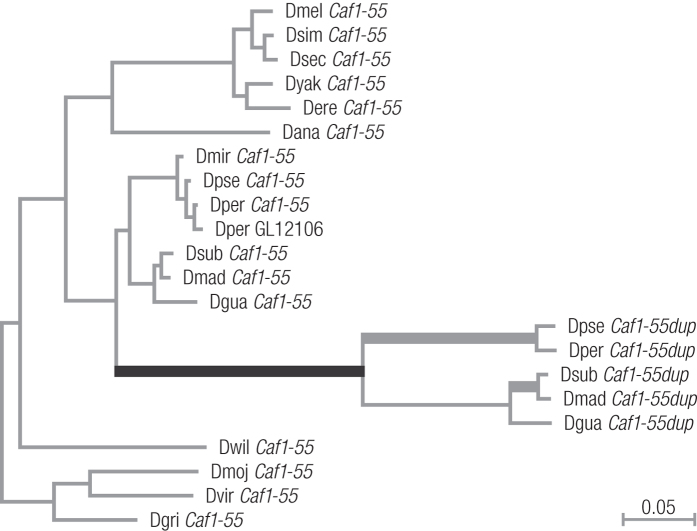
Phylogenetic tree based on the divergence of *Caf1-55* and *Caf1-55dup*. The three thick branches correspond to those branches with evidence of positive selection (*p*-value <0.05) according to the aBSREL random effects branch site method^21^. The thick black branch remains significant after correction for multiple testing. The scale in the lower right corner indicates nucleotide substitutions per site. Dmel = *D. melanogaster*, Dsim = *D. simulans*, Dsec = *D. sechellia*, Dyak = *D. yakuba*, Dere = *D. erecta*, Dana = *D. ananassae*, Dmir = *D.miranda*, Dpse = *D. pseudoobscura*, Dper = *D. persimilis*, Dsub = *D. subobscura*, Dmad = *D. madeirensis*, Dgua = *D. guanche*, Dwil = *D. willistoni*, Dmoj = *D. mojavensis*, Dvir = *D. virilis* and Dgri = *D. grimshawi*.

The maximum likelihood tree inferred from the multiple nucleotide alignment shows a strong increase in the substitution rate after the duplication event that gave rise to *Caf1-55dup* (Fig. 2). As a first step towards analyzing this result, three evolutionary branch models implemented in PAML^20^ were compared to detect the presence of lineages with a significant increase in the ω estimates (ω = d_N_/d_S_) in the phylogeny. The 3 R model, which assumes three different ω estimates (one for the branches of the ancestral *Caf1-55* gene, one for *Caf1-55dup* and one for the unique *D. persimilis* duplicate GL12106), fitted the data better than the null model M0, which assumes a single ω for all branches (*p* < 0.0001). Likewise, the null model M0 was rejected when compared with the FR model, which has a different ω for each branch (*p* < 0.0001). In contrast, the FR model did not explain the data better than 3 R when this was used as the null model (*p* = 0.1229). These results clearly indicate that the heterogeneity in ω values among branches is mainly due to differences in ω in the branches of the ancestral *Caf1-55, Caf1-55dup* and GL12106. In fact, ω_Caf1-55_ = 0.0012, ω_Caf1-55dup_ = 0.3787 and ω_GL12106_ = 1.3842. Different likelihood methods have been developed to infer whether increases in ω estimates in particular lineages can be explained by the action of positive selection in these lineages. The aBSREL random effects branch site method^21^ provided evidence of episodic selection in three *Caf1-55dup* lineages (Fig. 2): the *Caf1-55dup* lineage prior to the divergence of the obscura group species (*p* = 0.0037), the lineage prior to the divergence of *D. pseudoobscura* and *D. persimilis (p* = 0.0058) and the lineage prior to the divergence of *D. subobscura* and *D. madeirensis (p* = 0.0193). However, *p*-values remained significant only for the *Caf1-55dup* lineage that leads to the obscura species after correcting for multiple testing (*p* = 0.0412). These results were also confirmed by the branch site models implemented in PAML, showing positive selection in the lineage prior to the divergence of the obscura group species (*p* = 0.0002).

Although the aBSREL and PAML branch site models were developed to detect positive selection, any increase in ω estimates may also be due to relaxed purifying selection. The RELAX method^22^, which relies on the aBSREL random effects branch site model, was developed to distinguish between positive and relaxed selection. This method was applied to our data set, considering the *Caf1-55* branches as reference branches and the *Caf1-55dup* branch prior to the divergence of the obscura group species as the test branch. The action of positive diversifying selection was confirmed in this last lineage (*p* < 0.0001) with a selection intensity parameter (k) equal to 50. The Bayes Empirical Bayes (BEB) approach^23^ implemented in PAML identified 22 codons with a high posterior probability of having evolved under positive selection (see Supplementary Fig. S3) in *Caf1-55dup* after its origin by duplication. Most of these codons also show a rather high evidence ratio of having been the target of positive selection according to BUSTED^24^.

It is noteworthy that despite the strong acceleration in the substitution rate detected in the *Caf1-55dup* lineage after the duplication event, no effect in the *Caf1-55* lineage can be inferred by either the PAML or aBSREL likelihood methods. Therefore, there is no evidence of any relaxation of the purifying selection acting on the ancestral *Caf1-55* gene as a consequence of its duplication. In fact, there are no fixed amino acid differences in CAF1-55 between species of the obscura group and other *Drosophila* species (see Supplementary Fig. S3).

**Figure 3:**
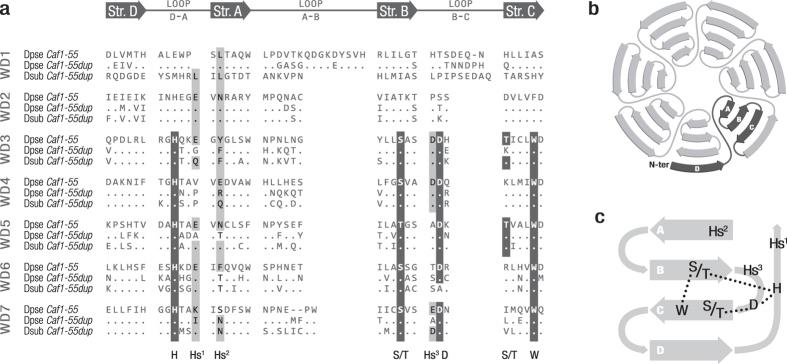
Comparison of the seven WD40 repeats between CAF1-55 and CAF1-55DUP. (**a**) Alignment of the seven WD40 repeats, which are composed by four β-strands (Str.) interspersed with loops, as represented graphically above the alignment. Loop C–D is not included due to its poor conservation and absence of relevant residues. The ancestral CAF1-55 protein is represented by the sequence of *D. pseudoobscura* and the CAF1-55DUP protein by the sequences of *D. pseudoobscura* and *D. subobscura*. Highlighted in black are conserved residues involved in the formation of hydrogen bonds (His, Ser/Thr, Asp, Trp) and highlighted in grey are hotspot residues (Hs^1–3^) implicated in protein–protein interactions (i.e., any of the binding-type amino acids: Arg, His, Lys, Asp, Glu, Trp, Tyr, Phe, Leu, Ile, Met, Asn or Gln). (**b**) Diagram of the general β-propeller structure. The first WD40 repeat is highlighted in black, showing that it is composed by the D strand of one blade and the A, B and C strands of the next blade. (**c**) Location of the key residues of each repeat over a β-propeller blade. Hydrogen bonds are represented by dotted lines.

The results of the WDSP^25^,^26^ analysis indicated that CAF1-55DUP contains the seven WD40 repeats present in CAF1-55. The average scores estimated by WDSP for the WD40 repeats of CAF1-55 are 86.70 and 86.57 in *D. melanogaster* and *D. pseudoobscura*, respectively. These scores for CAF1-55DUP are also high in the five species of the obscura group, ranging from 83.36 in *D. persimilis* to 87.48 in *D. subobscura*. The first repeat has the lowest score in both paralogs and is slightly displaced in the CAF1-55DUP protein of *D. subobscura, D. madeirensis* and *D. guanche* (see Supplementary Fig. S3). On the other hand, CAF1-55DUP contains 21 of the 22 residues that in CAF1-55 are involved in the formation of the hydrogen bonds that shape the four β-strands of each blade present in the seven-bladed β-propeller structure. However, the residues potentially implicated in protein–protein interactions (hotspots) are more divergent between the paralogs (Fig. 3). Residues that have evolved under positive selection according to the BEB and BUSTED methods are mainly located in the A–B or C–D loops and D β-strands (see Supplementary Fig. S3).

The duplication of *Caf1-55* in the obscura lineage would have occurred 18 Mya (95% highest posterior density (HPD) interval: 13–23 Mya) according to the BEAST 2 software^27^. This estimate is relative to the calibration points used in the analysis^28^, which assumed that the Drosophila and Sophophora subgenera split 32 Mya and that the melanogaster and obscura groups split 24 Mya. In addition, the origin of the additional *Caf1-55* duplication in the *D. persimilis* lineage would have taken place 0.35 Mya (95% HPD interval: 0.05–0.76 Mya). This estimate is consistent with the estimated 0.50 Myr of divergence between *D. pseudoobscura* and *D. persimilis*^29^.

### *Caf1-55* and *Caf1-55dup* nucleotide polymorphism in *D. subobscura*

Nucleotide polymorphism in *D. subobscura* was analyzed in *Caf1-55* and *Caf1-55dup*. The multiple alignment of the 14 *Caf1-55* and 16 *Caf1-55dup* sequences identified 85 and 95 nucleotide polymorphic sites, respectively (see Supplementary Fig. S4). A *Caf1-55dup* polymorphic site in line OF14 is a nonsense mutation that causes the loss of the last three amino acids in the encoded protein. Nucleotide diversity (π = 0.0082 and π = 0.0081 in *Caf1-55* and *Caf1-55dup*, respectively) is similar in both genes (Table 1). The pattern of variation indicates an excess of singletons (although not significant) as reflected by the negative sign of Tajima’s D^30^ statistic. This would be expected as *D. subobscura* shows a genomewide excess of low frequency variants likely due to a population expansion soon after the penultimate glacial period^31^. Levels of synonymous variation in the coding region are also similar in *Caf1-55* and its paralog *Caf1-55dup* (π_s_ = 0.0208 in *Caf1-55* and π_s_ = 0.0222 in *Caf1-55dup*). In contrast, both genes differ substantially in levels of nonsynonymous variation. In fact, no nonsynonymous polymorphism was detected in *Caf1-55*, whereas *Caf1-55dup* presents 12 polymorphisms (π_a_ = 0.0022) that affect the encoded protein (see Supplementary Fig. S4). This difference in nonsynonymous variation is also evident in the estimates of nonsynonymous divergence between *D. subobscura* and *D. guanche* using either K_a_ or the K_a_/K_s_ ratio (Table 1). These results clearly indicate much stronger functional constraints and thus purifying selection acting against nonsynonymous substitutions in *Caf1-55* than in *Caf1-55dup*.

The HKA test^32^ did not detect a decoupling between silent polymorphism and divergence when comparing *Caf1-55* and *Caf1-55dup* genes (χ^2^ = 0.4167, 1 df, *p* = 0.518) using *D. guanche* as the outgroup. On the other hand, the MK test^33^ was used independently for each gene (*Caf1-55* or *Caf1-55dup*) to detect a putative decoupling in the polymorphism to divergence ratio for synonymous and nonsynonymous mutations. The MK test rendered a significant result only in *Caf1-55dup*. In fact, the number of synonymous and nonsynonymous polymorphisms (24 and 12, respectively) and the number of synonymous and nonsynonymous fixed differences (21 and 30, respectively) differed significantly according to a χ^2^ test of independence (χ^2^ = 5.49, 1 df, *p* = 0.0191). This result and a neutrality index lower than 1 (NI = 0.350) indicate a significant excess of nonsynonymous substitutions fixed in *Caf1-55dup* during the divergence of *D. subobscura* and *D. guanche*. The fraction of adaptive amino acid substitutions estimated according to the α parameter^34^ is 0.650. The α parameter might be overestimated and even evidence of adaptive selection inferred by the MK test might be artifactual when nonsynonymous mutations are under weak selection and there are strong differences in effective size between the ancestral and current populations^35^,^36^. This is because slightly deleterious nonsynonymous mutations might have been fixed in a small ancestral population but they no longer segregate in a current large population. The demographic history of *D. subobscura* indicates that the species is under an expansion process^31^. However, no evidence of an important reduction in effective population size in the past was inferred. Therefore, it seems unlikely that the *D. subobscura* changes in effective size might have biased the results of the MK test.

## Discussion

Gene duplication is an important evolutionary mechanism to generate new genes and functions. Different models have been proposed to explain the origin, maintenance and evolution of gene duplicates^37^. In *Drosophila*, the most prevalent mechanism by which duplicated genes are retained is neofunctionalization^16^. Under neofunctionalization one gene is under purifying selection and retains the ancestral function, whereas the other gene acquires a new function by positive selection. The differential action of selection on both paralogs causes a strong asymmetry in nonsynonymous substitution rates^38^. Generally, the ancestral gene is the constrained copy that evolves under purifying selection and the new duplicate is the unconstrained copy that acquires a new function under positive selection^39^,^40^. The disruption in the new copy of the regulatory sequences, mainly of the 5′ flanking region, during the duplication process itself can explain how selection discriminates between the two paralogs^41^. Despite this general scenario, relaxation of purifying selection during divergence between paralogs cannot be ruled out either for the ancestral copy due to gene redundancy or the new gene.

The evolution of the *Caf1-55* and *Caf1-55dup* paralogs analyzed herein seems to have followed a neofunctionalization process. This process has been led by strong purifying selection acting on the ancestral copy (*Caf1-55*) and by positive selection on the new copy (*Caf1-55dup*). In fact, there is no evidence of relaxed selection in *Caf1-55* after the duplication, which means that selection could have discriminated between the redundant gene copies immediately after their origin. The *Caf1-55* duplication occurred via a DNA-mediated event and only affected *Caf1-55*, as those genes flanking *Caf1-55* are absent around *Caf1-55dup*. Moreover, the duplicated segment likely did not include the regulatory regions upstream from *Caf1-55* since sequence similarity when comparing *Caf1-55* and *Caf1-55dup* in the obscura species is restricted to the 5′ UTR. It is thus feasible that *Caf1-55dup* was not actually a redundant version of *Caf1-55* after its origin because it was poorly expressed or was not expressed at all. This argument, however, prompts the question why *Caf1-55dup* was maintained and fixed. It is possible that the expression level of *Caf1-55dup* increased only after the gene had accumulated enough mutations to ensure a difference in function between CAF1-55 and CAF1-55DUP. In contrast to the strong purifying selection acting on *Caf1-55*, there is clear evidence of the action of adaptive positive selection on *Caf1-55dup*. The adaptive process can be detected both in the lineage ancestral to the obscura group species (i.e., after the duplication), and more recently during the divergence of *D. subobscura* and *D. guanche*, as revealed by the MK test in the intra- and interspecific analysis. In addition, the intraspecific analysis indicated that purifying selection against nonsynonymous polymorphism is much stronger in *Caf1-55* than in *Caf1-55dup* (Table 1).

CAF1-55 is a subunit of the PRC2 Polycomb complex. This complex contains three additional proteins: E(Z), ESC and SU(Z)12. In a previous study^14^, it was shown that the genes coding these proteins suffered a significant increase in the nonsynonymous substitution rate in the lineage ancestral to the obscura group species. Therefore, in this lineage not only took place the duplication of *Caf1-55*, but also an acceleration in the fixation rate of nonsynonymous changes in the genes encoding proteins that interact with CAF1-55. The coincidence of both events in the same lineage might suggest that they are related. In fact, even after CAF1-55DUP accumulated some adaptive changes, CAF1-55 and CAF1-55DUP could have competed to be incorporated in protein complexes. Thus, it can be envisaged that the changes introduced in E(Z), ESC and SU(Z)12 could have prevented the misincorporation of CAF1-55DUP in PRC2 complexes or, alternatively, that they could have allowed the incorporation of CAF1-55DUP in PRC2 complexes if CAF1-55DUP had a Polycomb-related function.

**Figure 4:**
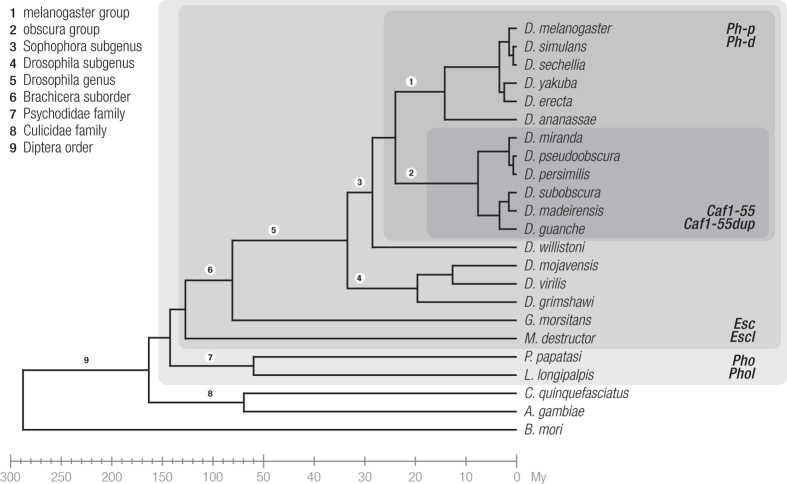
Duplication events of the Polycomb group genes in the Diptera phylogeny. Grey backgrounds group species that share the presence of a particular duplication. The intensity of the shadows ranges from very light grey, which indicates the most ancient duplication (*Pho*/*Phol*), light grey (*Esc*/*Escl* duplication), grey (*Ph-p*/*Ph-d* duplication) and dark grey, which shows the most recent duplication (*Caf1-55/Caf1-55dup*). The bar at the bottom indicates divergence times^28^,^55^ on two different scales. *G. morsitans = Glossina morsitans, M. destructor = Mayetiola destructor, P. papatasi = Phlebotomus papatasi, L. longipalpis = Lutzomyia longipalpis, C. quinquefasciatus = Culex quinquefasciatus, A. gambiae = Anopheles gambiae* and *B. mori = Bombix mori*.

*Caf1-55* enlarges the number of PcG genes duplicated in *Drosophila*. PcG genes code important epigenetic regulators and are mainly single copy genes in the *Drosophila* genus. In fact, PcG proteins form repressive complexes and thus the duplication of PcG genes may hinder maintenance of the proper stoichiometry between the interacting subunits of a protein complex. However, three additional PcG gene duplications have been reported in at least some *Drosophila* species: *Pho*/*Phol (Pleiohomeotic* and *Pleiohomeotic like*), *Esc*/*Escl (Extra sexcombs* and *Extra sexcombs like*) and *Ph-p*/*Ph-d (Polyhomeotic proximal Polyhomeotic distal*). A search in the genomes of different Diptera species using *Bombyx mori* as the outgroup was performed to gain a better insight into the duplication events affecting PcG genes and to date the detected duplications (Fig. 4). *Pho* and *Phol* are the most ancient duplicates (~150 Mya) as both paralogs are present in all Diptera analyzed except the two members of the Culicidae family. The *Esc*/*Escl* duplication took place later, after the split of the Psychodidae family (~130 Mya). The duplication *Ph-p*/*Ph-d* is more recent (~30 Mya) as the presence of both copies is only shared by the species of the Sophophora subgenus, with the exception of *D. willistoni*. Therefore, the *Caf1-55*/*Caf1-55dup* duplication in the ancestral branch of the obscura group species included in the Sophophora subgenus is the most recent and took place ~18 Mya. The lack of *Caf1-55dup* in *D. melanogaster* likely contributed to the fact that this duplication has remained undetected until now.

The evolutionary fate of the derived duplicate differs in the four duplicated PcG genes as reflected in the maximum likelihood phylogenetic trees inferred from divergence among paralogs (see Supplementary Fig. S5). As stated above, *Caf1-55dup* most likely underwent a neofunctionalization process. According to its phylogenetic tree, a similar process seems to have affected *Phol* after its origin via an RNA-mediated duplication. *Phol* neofunctionalization is also supported by ChIP on chip experiments that indicate that the binding patterns of PHO and PHOL do not always overlap in the genome^42^, although both proteins bind to the same target DNA sequence *in vitro*. In contrast, *Ph-p* and *Ph-d* are paralogous tandem genes that are known to have evolved under gene conversion^43^, which is consistent with the clustering of genes and species in the reconstructed phylogenetic tree. The single *Ph* gene present in the Drosophila subgenus species and in *D. willistoni* has a structure more similar to *Ph-p* than to *Ph-d*, suggesting that *Ph-p* is the ancestral gene. However, although the coding regions of *Ph-d* and *Ph-p* are very similar due to gene conversion, their regulatory regions are entirely different, suggesting functional divergence at the expression level. Finally, the *Esc*/*Escl* phylogenetic tree indicates that *Escl* is the ancestral gene and *Esc* the derived duplicate, as already suggested by Ohno *et al*.^44^. However, the divergence between paralogs after the duplication event is much lower than in the case of *Phol* and *Caf1-55dup*. ESC and ESCL, like CAF1-55, are members of the WD40 protein family and both can be incorporated alternatively in the PRC2 Polycomb complex. Nevertheless, ESC is present at high levels during embryogenesis, and ESC-containing PRC2 complexes are critical during early development, whereas the peak abundance of ESCL is found during postembryonic stages^45^. Therefore, the differential expression of the two paralogs during development would suggest a neofunctionalization process mainly at the expression level.

The *Esc*/*Escl, Pho*/*Phol* and *Ph-d*/*Ph-p* paralogs are therefore not strictly redundant, although they code Polycomb proteins with related functions. The results obtained by WDSP indicate that CAF1-55DUP is a member of the WD40 protein family, which suggests that it has retained its function as a scaffold to facilitate the interaction between proteins. However, WD40 is one of the most widespread protein families in eukaryotic organisms and WD40 domains are among the most versatile interactors^9^. Therefore, it is not clear whether CAF1-55DUP has a PcG-related function. In fact, although the residues implicated in the formation of hydrogen bonds are highly conserved between CAF1-55 and CAF1-55DUP, the residues that confer the capacity for and specificity of protein–protein interactions are more divergent. The decoupled conservation of these two kinds of residues could indicate that the general function of WD40 is maintained in both paralogs but that they probably interact with different proteins, supporting the neofunctionalization hypothesis.

In addition, the RNA-seq data of *D. pseudoobscura*^46^ available in FlyBase show that *Caf1-55* and *Caf1-55dup* differ in their expression profiles. *Caf1-55* is ubiquitously expressed: at moderate levels in carcass and head, and at high and very high levels in testis and ovary, respectively. In contrast, the expression levels of *Caf1-55dup* are much lower, being moderate only in the testis and ovary (see Supplementary Fig. S6). Functional experiments would be required to characterize the function of *Caf1-55dup* and to explain its differential expression profile.

In summary, the gene encoding the multifunctional and highly conserved protein CAF1-55 is duplicated in the *Drosophila* species of the obscura group. This duplication took place ~18 Mya and enlarges the number of PcG-duplicated genes. The duplicates have suffered clear neofunctionalization, with the action of strong purifying selection on the ancestral copy and of positive selection on the new copy. Positive selection has also acted in a more recent timescale during the divergence of *D. subobscura* and *D. guanche*, as reflected in the *D. subobscura* intraspecific analysis. However, CAF1-55DUP has retained the functional domains of CAF1-55 in all obscura species, suggesting that it is also a member of the WD40 protein family. Given that proteins of this family are among the most versatile interactors, it is not clear whether CAF1-55DUP has a PcG-related function.

## Material and Methods

### Fly stocks and sequencing

The *chcu* strain of *D. subobscura* and highly inbred lines of *D. madeirensis* and *D. guanche* were used to sequence *Caf1-55dup* in these species. Nucleotide polymorphism in *Caf1-55* and *Caf1-55dup* was analyzed in highly inbred lines of *D. subobscura* obtained as described in Pratdesaba *et al*.^31^ after sampling a natural population of the species in the Observatori Fabra (Barcelona, Catalonia, Spain). Lines to be studied were chosen by taking into account the chromosomal location of both genes, as inferred by *in situ* hybridization on polytene chromosomes using biotinylated probes^47^. Thus, the selected lines that had either the O_st_ or O_3+4+8_ arrangements, although differing by three overlapping inversions, are homokaryotypic for the proximal half of the O chromosome where *Caf1-55* and *Caf1-55dup* map. Therefore, no effect of the extensive *D. subobscura* inversion polymorphism is expected on the level and pattern of nucleotide variation detected in *Caf1-55* and *Caf1-55dup*. Genomic DNA of these fly stocks, as well as of *D. pseudoobscura, D. persimilis* and *D. miranda*, was available in our laboratory.

The *Caf1-55* and *Caf1-55dup* genes were PCR amplified with primers designed according to the *D. pseudoobscura* sequence using the OLIGO program^48^. Amplicons were purified with Multiscreen plates (Millipore) and both strands were completely sequenced with the ABI Prism BigDye Terminators 3.0 Cycle kit (Applied Biosystems) using internal primers. Sequencing reactions were run on an ABI PRISM 3700 sequencer. Partial sequences were assembled using the SEQMAN program of the LASERGENE package^49^. Total RNA of the obscura species was extracted with the RNeasy™ Mini Kit (Qiagen) and then the cDNA was synthesized using the SuperScript™ III Reverse Transcriptase Kit (Thermo Fisher Scientific), following in both cases the manufacturers’ instructions. Subsequently, *Caf1-55dup* cDNA was PCR amplified and sequenced as explained above. The sequences of the primers used in the PCR amplification and sequencing, as well as the PCR conditions, are available in the electronic supplementary material, Table S1.

### Divergence analysis

For the species sequenced by the *Drosophila* 12 Genomes Consortium^15^ and *D. miranda*, the sequences of *Caf1-55* and its paralogs were retrieved from FlyBase after BLASTN searches using the default parameters. Sequence similarity and synteny conservation with flanking genes were analyzed to distinguish between ancestral and derived gene copies. The *Caf1-55* sequences of *D. subobscura, D. madeirensis* and *D. guanche* were retrieved from the EMBL Nucleotide Sequence Database (accession numbers LN864767-69) and those of *Caf1-55dup* were determined in the present study. Orthologous and paralogous sequences were multiply aligned using the MUSCLE program^50^ implemented in the MEGA6 software package^51^ according to the alignment of the predicted proteins.

Amino acid conservation along the multiple alignment was inferred for each gene independently using the Clustal X program^52^, which assigns a conservation score for each position of the alignment based on the mean of the distances between codons (according to weight matrix BLOSUM62) and normalized by the percentage of sequences without gaps at this position. The maximum likelihood approach implemented in MEGA6 was used to infer the branch lengths of the accepted phylogenetic tree of the studied species based on nucleotide divergence according to the GTR (general time reversible) model.

The PAML v4 package^20^ was used to compare alternative evolutionary branch models that differ by assumptions concerning the ω estimates (ω = d_N_/d_S_, where d_N_ corresponds to nonsynonymous and d_S_ to synonymous divergence). The M0 model assumes a single ω estimate for all branches, the 3 R model assumes three different ω estimates, each one for a different set of branches, and the FR model assumes a different ω for each branch. The branch-site test of positive selection (test 2 in Zhang *et al*.^53^) implemented in the same package was performed to detect the putative presence of codons under positive selection (ω > 1) in particular branches predefined as foreground branches. The Bayes Empirical Bayes (BEB) method^23^ also implemented in PAML was used to identify the sites with a high posterior probability of having evolved under positive selection in these branches (ω > 1). In addition, the data set was analyzed by three methods implemented in the HYPHY software package^54^. First, the aBSREL branch-site random effects likelihood method^21^ was used to detect evidence of positive selection in particular branches. This method, in contrast to the branch-site methods implemented in PAML, does not require to predefine foreground branches in the phylogeny. Second, the RELAX approach^22^ was used to further confirm that increases in ω estimates were indeed due to positive selection and not to relaxed selection. Finally, the BUSTED approach^24^ was applied to identify the codon sites under positive selection in foreground branches.

The software WDSP^25^,^26^ was used to determine whether the protein CAF1-55DUP retains the WD40 repeats structure present in CAF1-55. WDSP infers the secondary structure of a given protein, identifies WD40 repeats and estimates a score for each detected WD40 repeat. The tested protein is considered a member of the WD40 family when it presents more than six repeats and the average score of these repeats is greater than 48.

*Caf1-55* is not the only duplicated Polycomb gene in *Drosophila*. In fact, other PcG genes are known to be duplicated in *D. melanogaster: Ph-p*/*Ph-d, Esc*/*Escl* and *Pho*/*Phol*. The OrthoDB hierarchical catalog of orthologous genes (http://orthodb.org) was used to infer the presence or absence of these genes in other non-*Drosophila* insect species. The phylogenetic relationships of the insect species in which both paralogs are present and the timing of divergence reported in Misof *et al*.^55^ were used in this analysis.

Estimates of the duplication events of *Caf1-55* were inferred using the BEAST 2 (Bayesian Evolutionary Analysis by Sampling Trees) software platform^27^. The analysis was performed according to a lognormal relaxed clock and the GTR substitution model. The divergence dates of the *Drosophila* species based on the mutation rate^28^ were used as calibration points. The MCMC analysis was run with a chain length of 100 million steps, sampling every 10 000 steps.

### Nucleotide polymorphism analysis

The assembled sequences of each *D. subobscura* line were aligned using the MUSCLE program^50^. Levels of nucleotide polymorphism were estimated by standard parameters, such as the number of polymorphic sites (S) and nucleotide diversity (π). In coding regions, π was estimated independently for synonymous (π_s_) and nonsynonymous (π_a_) variation. The pattern of variation was analyzed using the Tajima’s D^30^ statistic. The HKA^32^ and MK^33^ tests were performed to detect a putative decoupling of polymorphism and divergence levels either at silent (noncoding and synonymous) sites between the two genes (HKA test) or between synonymous and nonsynonymous sites of the same gene (MK test). The DnaSP v5 program^56^ was used to perform most of the polymorphism analyses and the HKA program^57^ to perform this neutrality test.

## Additional Information

**Accession codes:** The newly reported sequences are deposited in the EMBL/GenBank Data Libraries under accession numbers: LT600471 to LT600503

**How to cite this article**: Calvo-Martín, J. M. *et al*. Evidence of neofunctionalization after the duplication of the highly conserved Polycomb group gene *Caf1-55* in the obscura group of *Drosophila. Sci. Rep.* **7**, 40536; doi: 10.1038/srep40536 (2017).

**Publisher's note:** Springer Nature remains neutral with regard to jurisdictional claims in published maps and institutional affiliations.

## Supplementary Material

Supplementary Information

## Figures and Tables

**Table 1:** Estimates of nucleotide polymorphism in *D. subobscura* and of divergence between *D. subobscura* and *D. guanche* corrected for multiple hits.

	*Caf1-55*	*Caf1-55dup*
number of sequences	14	16
number of sites	2658	2504
number of polymorphic sites (S)	85	95
number of singletons	46	58
nucleotide diversity (π)	0.0082	0.0081
Tajima’s D	−0.8618	−1.3430
synonymous diversity (π_s_)	0.0208	0.0222
nonsynonymous diversity (π_a_)	0	0.0022
synonymous divergence (K_s_)	0.1417	0.1009
nonsynonymous divergence (K_a_)	0.0010	0.0327
K_a_/K_s_	0.0070	0.3238
