# Supplementary material for: Evidence of neofunctionalization after the duplication of the highly conserved Polycomb group gene Caf1-55 in the obscura group of Drosophila
Source: Sci Rep. 2017 Jan 17;7:40536. doi: 10.1038/srep40536 (PMC5240099; doi:10.1038/srep40536)
Supplement: Supplementary Information [file srep40536-s1.pdf]

Supplementary Information

Evidence of neofunctionalization after the duplication of the highly conserved Polycomb group gene *Caf1-55* in the obscura group of *Drosophila*

Juan M. Calvo-Martín, Montserrat Papaceit and Carmen Segarra

a

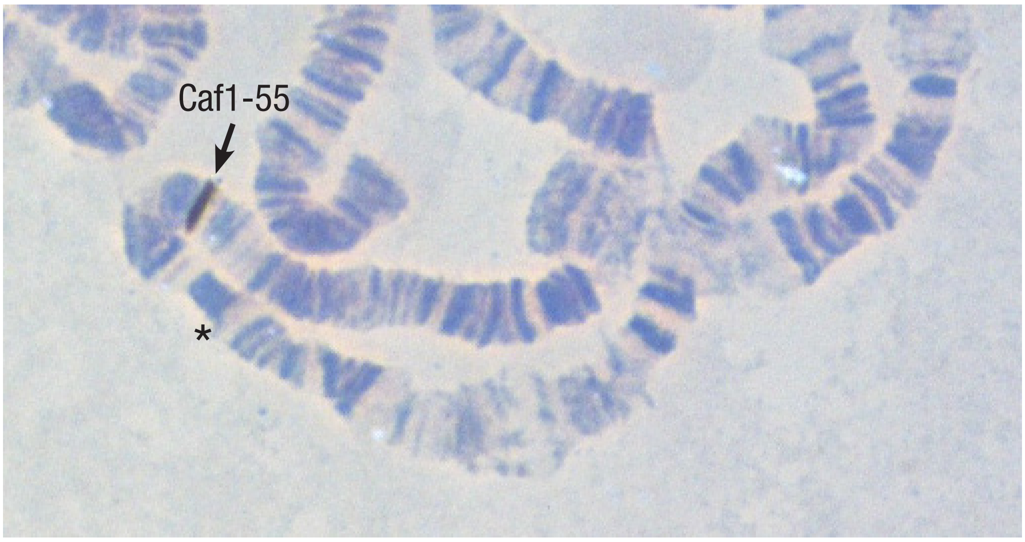

b

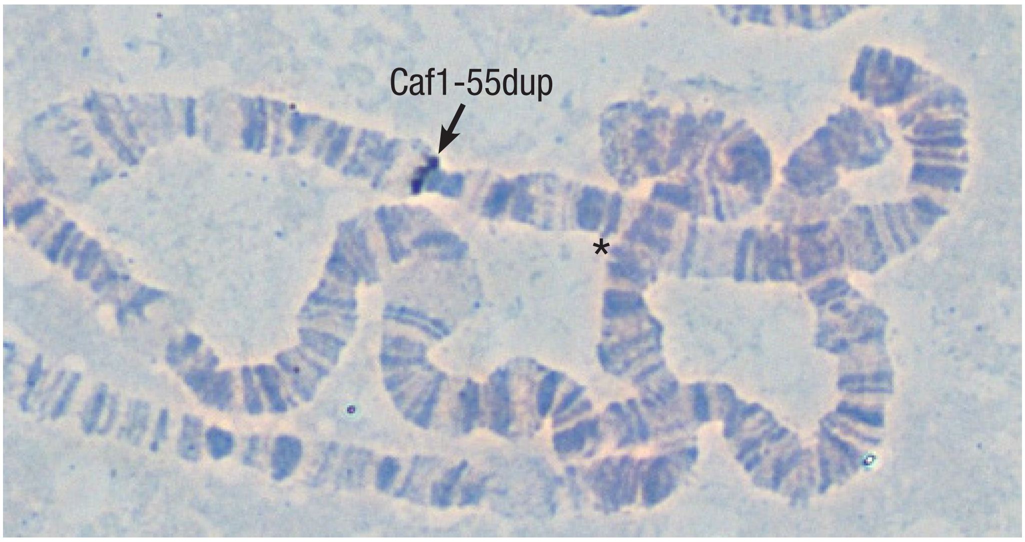

Figure S1. Location of *Caf1-55* (A) and *Caf1-55dup* (B) by *in situ* hybridization on *D. subobscura* polytene chromosomes. Arrows indicate the position of each gene according to the hybridization site of the biotinylated probes and asterisks the position of its paralog. *Caf1-55* and *Caf1-55dup* are located in the cytogenetic bands 79C and 80D, respectively.

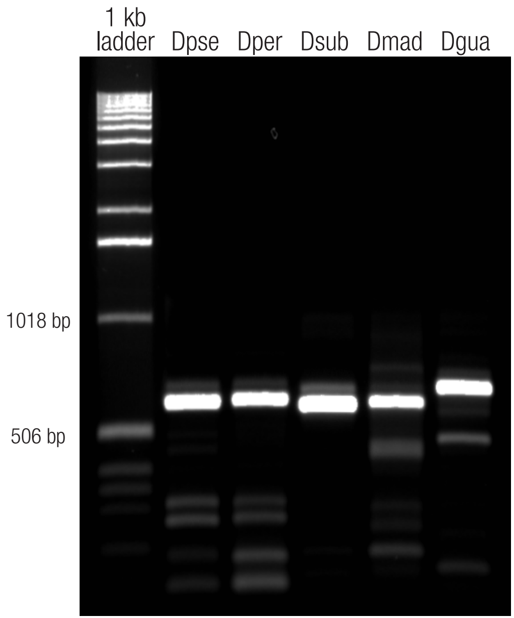

Figure S2. PCR amplification of the second and third exons of *Caf1-55dup* using the cDNA of five Drosophila species of the obscura group as template. Fragment sizes and posterior sequencing confirm that the recovered and amplified cDNA proceed from processed mRNA. Species names as in Figure 2.

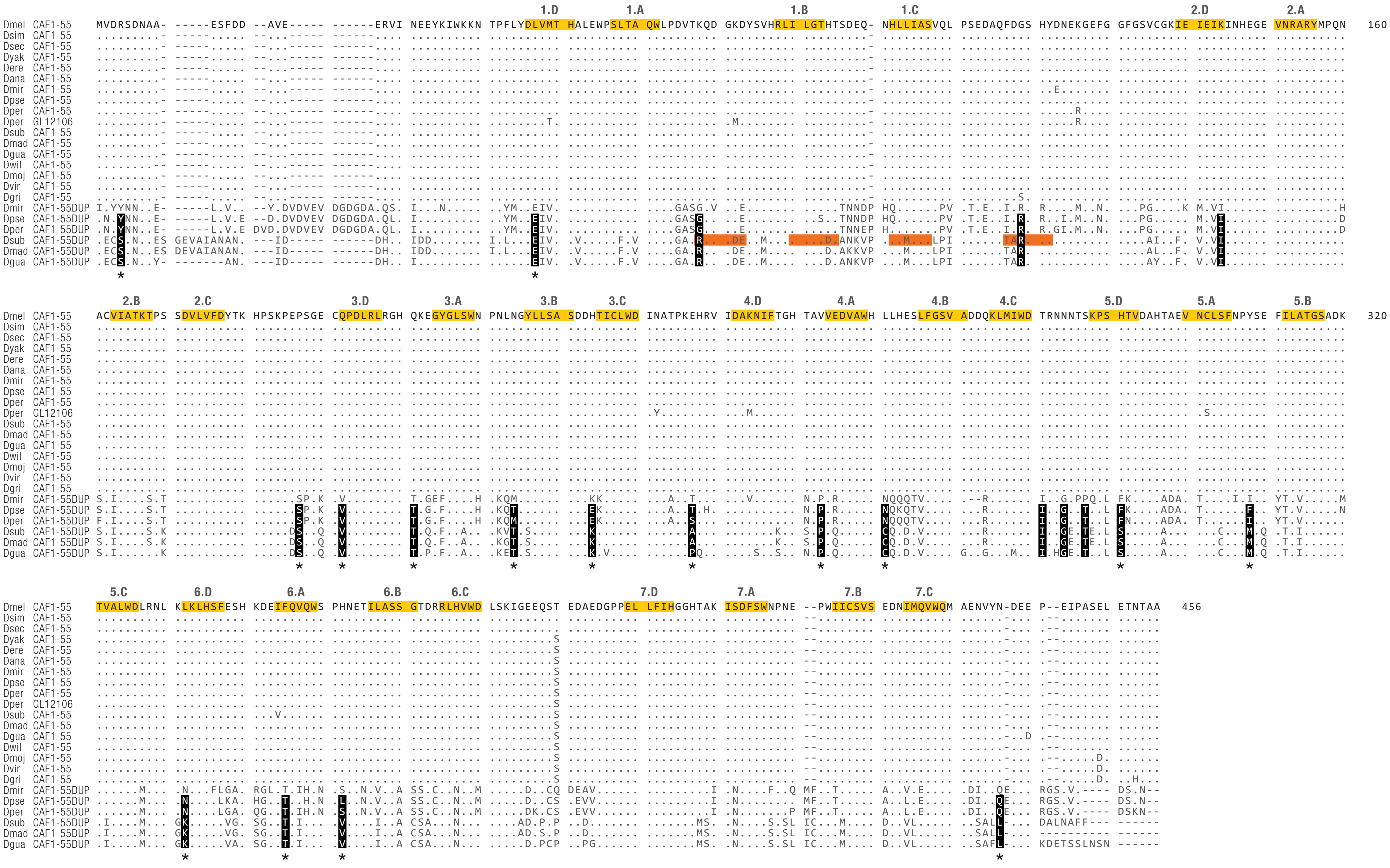

Figure S3. Multiple alignment of CAF1-55, CAF1-55DUP and the private *D. persimilis* duplicate GL12106. Dots represent the same amino acid as in the *D. melanogaster* sequence used as reference and dashes represent alignment gaps. Sites labeled with a black background are those corresponding to codons that present a high posterior probability of having evolved under positive selection according to the BEB method. Among these sites, those labeled with an asterisk were also detected as positively selected by the BUSTED method. Selected sites are not indicated in the CAF1-55DUP sequence of *D. miranda* as this sequence was not included in the selection analysis (see text). Species names as in Figure 2. Shadowed in yellow are the β-strands of each of the 7 WD40 repeats identified by the program WDSP. Shadowed in orange are the predicted β-strands of CAF1-55DUP first repeat in *D. subobscura*, which are maintained in *D. madeirensis* and *D. guanche*, and are slightly displaced relative to the other protein sequences.

Caf1-55

5' | I1 | E2 | I2 | E3 | 3'

Line	34	55	75	88	123	139	142	145	155	172	180	195	220	244	247	273	284	304	314	329	346	348	361	372	374	409	411	414	434	435	438	446	475	478	479	483	494	535	537	571	579	602	640	641	660	687	739	767	812	818	832	919	960	968	1150	1294	1375	1456	1535	1543	1546	1572	1614	1623	1629	1686	1752	1896	1951	1980	1986	2004	2013	2016	2028	2067	2082	2271	2298	2350	2358	2413	2453	2461	2465	2490	2497	2509	2510	2551	2557	2611	2618
OF01	C	G	C	G	T	A	C	A	G	T	A	A	A	C	C	C	G	A	T	C	C	-	A	A	C	C	C	A	T	C	C	C	G	A	T	T	G	C	T	G	C	$(A)_{12}$	T	-	A	A	A	A	G	A	C	G	T	C	G	G	C	A	G	G	G	T	C	T	T	C	C	A	T	G	C	C	G	C	C	G	A	C	C	T	T	T	G	A	A	T	-	C	C	C	G	C	A
OF07	.	.	.	.	.	.	A	d_8	.	G	.	.	C	.	.	T	.	T	.	.	.	i_3	.	.	.	A	A	d_3	A	.	.	.	.	.	A	.	.	.	.	.	A	$(A)_{12}$	.	-	.	C	.	.	.	.	.	.	.	.	.	.	T	C	.	T	.	.	T	C	C	.	T	G	.	.	.	.	.	.	T	.	.	.	.	.	C	.	.	.	.	.	-	.	.	.	C	T	T
OF14	.	.	.	.	.	.	A	d_8	.	G	.	.	C	.	.	T	.	T	.	.	.	i_3	.	.	.	A	A	d_3	A	.	.	.	.	.	A	.	.	.	.	.	A	$(A)_{12}$	.	-	.	C	.	.	.	.	.	.	.	.	.	.	.	.	.	.	.	.	.	.	.	.	.	.	.	.	.	.	.	.	.	.	.	.	.	.	C	A	.	.	.	C	-	.	.	T	C	.	.
OF16	T	A	.	.	.	.	.	.	.	G	.	T	.	.	.	.	.	.	.	.	.	-	.	.	.	.	.	.	G	.	.	.	A	G	A	G	.	G	C	.	A	$(A)_{15}$	C	A	G	.	G	T	.	G	.	.	.	.	T	.	T	C	.	.	.	.	T	.	C	.	.	G	.	.	T	.	.	.	.	.	G	.	.	.	C	A	.	.	.	.	-	.	.	T	C	.	.
OF18	.	.	T	.	.	G	A	.	.	G	.	.	C	.	.	T	.	T	.	.	.	i_3	.	.	A	A	A	.	A	.	.	G	.	.	A	.	.	.	.	A	.	$(A)_{12}$	.	-	.	.	.	.	.	.	.	.	.	.	.	A	.	C	.	.	.	C	T	.	C	.	.	G	.	.	T	.	.	.	.	.	G	.	.	.	.	.	.	.	.	.	-	G	.	T	C	.	.
OF19	.	.	.	T	.	.	A	.	.	G	.	.	C	.	.	T	.	T	.	A	.	i_3	.	.	.	A	A	d_3	A	.	.	.	.	.	A	.	.	.	.	.	A	$(A)_{12}$	.	-	.	C	.	.	.	.	.	.	.	.	.	.	T	C	A	.	C	.	.	.	.	.	.	.	.	.	.	.	.	.	.	.	.	A	.	.	C	A	.	.	.	.	-	.	.	.	C	.	.
OF28	T	A	.	.	.	.	A	.	.	G	.	T	.	.	.	.	.	.	.	.	.	-	.	.	.	.	.	.	G	T	-	.	.	G	A	C	.	G	C	.	A	$(A)_{16}$	C	A	G	.	G	T	.	G	.	.	.	A	.	.	T	C	.	.	.	.	T	C	C	T	T	G	C	A	.	.	.	.	.	.	G	.	.	.	C	A	.	.	.	.	-	.	.	.	C	.	.
OF31	.	.	.	.	A	.	A	.	A	G	T	.	.	A	T	T	T	T	C	.	T	-	T	.	.	A	A	.	A	.	T	.	.	.	A	.	A	.	.	.	A	$(A)_{15}$	.	-	.	.	.	.	C	.	.	A	.	A	.	.	T	C	.	.	.	.	T	.	C	.	.	G	.	.	.	.	.	A	.	.	.	.	T	.	C	A	.	.	.	.	-	.	.	.	C	.	.
OF44	T	A	.	.	.	.	A	.	.	G	.	T	.	.	.	.	.	.	.	.	.	-	.	.	.	.	.	.	G	.	.	.	.	G	A	G	.	G	C	.	A	$(A)_{14}$	C	A	G	.	G	T	.	G	d_9	.	.	.	.	.	T	C	.	.	.	.	T	.	C	.	.	.	.	.	.	.	A	.	.	.	G	.	.	.	C	A	.	.	.	.	-	.	.	T	C	.	.
OF47	.	.	.	.	.	.	A	d_8	.	G	.	.	C	.	.	T	.	T	.	.	.	i_3	.	.	.	A	A	d_3	A	.	.	.	.	.	A	.	.	.	.	.	A	$(A)_{12}$	.	-	.	C	.	.	.	.	.	.	C	.	.	.	.	.	.	.	.	.	.	.	.	.	.	.	.	.	.	.	.	.	.	.	.	A	.	.	C	A	.	.	.	.	-	.	.	.	C	.	.
OF54	.	.	.	.	.	.	A	d_8	.	G	.	.	C	.	.	T	.	T	.	.	.	i_3	.	.	.	A	A	d_3	A	.	.	.	.	.	A	.	.	.	.	.	A	$(A)_{12}$	.	-	.	C	.	.	.	.	.	.	.	.	.	.	.	.	.	.	.	.	T	.	C	.	.	G	.	.	.	T	.	.	.	.	.	.	.	.	C	A	A	.	.	.	A	.	G	T	C	.	.
OF58	.	.	.	.	.	.	A	d_8	.	G	.	.	C	.	.	T	.	T	.	.	.	i_3	.	.	.	A	A	d_3	A	.	.	.	.	.	A	.	.	.	.	.	A	$(A)_{12}$	.	-	.	C	.	.	.	.	.	.	.	.	.	.	.	.	.	.	.	.	.	.	.	.	.	.	.	.	T	.	.	.	.	.	.	.	.	.	C	.	.	G	T	.	-	.	.	.	C	.	.
OF60	.	.	.	T	.	.	A	.	.	G	.	.	C	.	.	T	.	T	.	A	.	i_3	.	.	.	A	A	d_3	A	.	.	.	.	.	A	.	.	.	.	.	A	$(A)_{12}$	.	-	.	C	.	.	.	.	.	.	.	.	.	.	T	C	A	.	C	.	.	.	.	.	.	.	.	.	.	.	.	.	.	.	.	A	.	.	C	A	.	.	.	.	-	.	.	.	C	.	.
OF78	.	.	.	.	.	.	A	.	.	G	.	.	C	.	.	T	.	T	.	.	.	i_3	.	T	.	A	A	d_3	A	.	.	.	.	.	A	.	.	.	.	.	A	$(A)_{12}$	.	-	.	C	.	.	.	.	.	.	.	.	.	.	.	C	.	.	.	.	T	.	C	.	.	.	.	.	.	.	.	.	.	A	.	.	.	G	C	A	.	.	.	.	-	.	.	.	C	.	.
Dmad	.	A	.	.	.	.	A	.	.	.	.	.	.	.	A	T	.	T	.	.	.	-	T	.	.	A	A	.	A	.	.	.	.	G	A	.	.	G	C	.	A	$(A)_{12}$	C	A	G	.	G	.	C	.	.	A	.	.	.	.	.	T	.	.	.	A	.	.	.	.	T	G	C	.	.	.	.	.	.	.	.	A	.	.	C	.	.	.	.	.	-	.	.	.	C	.	.
Dgua	?	?	?	?	?	?	?	?	?	?	?	?	?	?	?	?	?	?	?	?	?	?	?	?	?	?	?	?	?	?	?	?	?	?	?	?	?	?	?	?	?	$(A)_{10}$	.	A	G	.	G	.	.	.	.	.	.	G	T	.	.	C	A	.	T	.	.	.	.	.	T	G	.	.	.	G	.	.	.	.	.	A	.	.	C	.	.	.	.	C	-	.	.	.	C	.	.

Caf1-55dup

5' | I1 | E2 | I2 | E3 | 3'

Line	34	37	52	86	154	171	197	249	251	259	262	272	276	282	313	340	377	392	398	400	408	409	433	484	498	506	509	545	549	564	565	578	598	613	615	627	692	702	725	727	755	756	769	786	795	855	896	908	917	921	945	1002	1062	1077	1149	1197	1239	1257	1272	1304	1315	1331	1332	1334	1370	1374	1459	1492	1555	1591	1611	1651	1678	1679	1730	1778	1831	1945	1951	1968	1981	2062	2073	2119	2121	2134	2150	2201	2245	2296	2302	2330	2389	2403	2413	2452	2454	2456	2462	2466	2485	2490
																																									*		*				*	*	*											*										*	*			*	*	*				*			+																			
OF01	T	T	i_6	G	G	G	A	G	A	T	A	G	G	T	G	A	A	A	A	A	A	A	G	G	$(A)_6$	A	G	T	A	C	T	T	G	G	C	T	T	A	C	C	T	T	A	C	T	A	C	G	A	G	A	C	T	C	T	C	C	G	G	G	C	G	-	C	T	T	T	T	T	G	T	C	C	A	A	C	C	C	A	T	C	A	T	G	-	T	T	T	G	T	T	A	C	T	T	T	T	G	C	C	A	A
OF07	C	C	i_6	.	.	.	T	.	.	.	.	.	A	A	A	.	.	.	.	C	.	C	.	.	$(A)_7$	.	T	.	.	.	.	.	.	.	.	.	.	.	.	T	C	C	.	.	.	.	.	.	.	.	.	.	C	.	.	.	.	.	.	.	.	A	-	T	C	.	C	.	.	.	C	T	.	.	.	.	.	.	G	.	T	.	.	.	-	.	.	C	.	.	C	.	.	A	.	A	.	T	.	.	.	.
OF14	.	.	i_6	.	.	.	T	.	.	.	.	.	.	A	A	.	.	.	.	.	.	.	.	.	$(A)_8$	.	.	.	.	.	.	.	A	.	.	.	.	.	.	T	C	C	.	.	.	G	.	.	.	.	.	.	.	.	.	T	.	.	.	.	.	A	A	.	C	.	.	.	.	.	C	.	.	.	.	.	.	.	.	.	.	C	G	.	-	.	.	C	.	C	.	.	.	.	.	.	.	.	.	.	.	.
OF16	C	.	i_6	.	.	.	T	.	.	.	.	.	.	A	A	.	.	.	.	C	.	.	T	.	$(A)_7$	.	.	.	.	.	.	.	.	.	T	.	.	.	.	T	C	C	.	.	.	G	.	.	.	A	.	.	.	.	G	.	.	T	.	.	.	A	A	.	C	.	.	.	.	.	C	T	.	.	.	.	.	.	.	.	.	C	.	.	-	.	.	C	.	.	.	.	.	.	.	.	.	.	.	.	.	.
OF18	.	.	i_6	A	.	.	T	.	.	.	.	T	.	A	A	.	T	.	G	C	.	.	.	A	$(A)_7$	T	T	.	.	.	.	C	.	.	.	.	.	.	.	T	C	C	C	.	.	G	.	.	.	.	.	.	.	.	.	.	.	.	.	.	.	.	-	.	C	.	.	.	.	.	C	.	.	.	.	.	.	.	.	.	.	C	.	A	i_2	.	.	C	.	.	C	.	.	A	.	.	.	.	.	.	.	.
OF19	.	.	i_6	.	.	.	T	.	T	.	.	.	.	A	A	.	.	.	.	C	.	.	.	A	$(A)_7$	.	.	A	.	.	.	.	.	.	.	.	.	.	.	T	.	.	.	.	.	.	.	.	.	.	.	.	C	.	.	.	.	.	.	.	.	A	-	.	C	C	.	.	.	.	C	.	.	.	.	.	T	.	.	.	.	.	.	.	-	.	.	C	.	.	.	.	.	.	.	.	G	.	G	T	.	.
OF21	.	.	-	.	.	T	T	.	.	.	.	.	.	A	A	.	.	.	.	C	.	C	.	.	$(A)_7$	.	.	A	.	.	.	.	.	.	.	C	.	.	.	T	C	C	.	.	.	.	.	.	.	.	.	.	.	.	.	.	.	.	.	.	.	A	-	.	C	.	.	.	.	.	C	.	.	.	C	.	.	.	.	.	.	.	.	.	-	.	.	C	.	.	.	.	.	.	.	.	G	.	G	T	.	.
OF28	C	.	i_6	.	.	.	T	.	.	.	.	.	.	A	A	.	.	.	.	C	.	.	.	.	$(A)_8$	.	.	.	.	.	.	.	.	.	.	.	.	.	.	T	C	C	.	.	.	G	.	.	.	.	.	T	.	.	.	.	.	.	.	A	.	.	A	-	.	C	.	.	A	.	.	C	.	.	.	.	.	.	.	.	.	.	.	.	-	.	G	C	.	C	.	.	.	.	.	.	G	.	.	.	.	T
OF31	.	.	i_6	.	.	.	T	.	.	.	.	.	.	A	A	.	.	.	.	C	.	C	.	A	$(A)_7$	.	.	.	.	.	.	.	.	.	.	.	.	C	.	T	.	.	.	.	.	G	.	.	.	.	.	.	.	.	G	.	.	.	.	.	.	A	-	.	C	.	C	.	C	.	.	.	A	.	.	.	.	T	.	.	.	C	.	.	-	.	.	C	.	.	.	.	.	.	.	.	G	.	.	.	.	T
OF40	.	.	i_6	.	.	.	T	.	.	.	T	.	.	.	A	.	.	.	.	C	.	C	.	.	$(A)_7$	.	.	A	.	.	.	.	.	.	.	.	.	.	G	T	C	C	.	.	.	G	.	.	T	.	.	.	C	.	.	.	T	.	.	T	A	A	A	.	C	.	C	.	.	.	C	T	.	.	.	G	.	.	.	A	.	C	.	.	-	.	.	C	.	.	.	.	.	.	C	.	.	.	.	.	.	T
OF44	.	.	i_6	.	.	.	T	.	.	.	.	.	.	A	A	.	.	.	.	C	.	C	.	.	$(A)_6$	.	.	.	.	.	.	.	.	.	.	.	.	.	.	T	C	C	.	.	.	G	.	.	.	.	.	.	C	.	.	.	.	.	.	.	.	A	-	.	C	.	C	.	.	.	C	.	.	.	C	.	.	.	.	.	.	.	.	.	-	.	.	C	.	C	.	.	.	.	.	.	.	.	.	.	C	T
OF47	.	.	i_6	.	.	.	T	.	.	C	.	.	.	A	A	.	.	T	.	C	.	.	.	A	$(A)_7$	.	.	.	.	.	.	.	.	.	.	.	.	C	.	T	.	.	.	.	.	G	.	.	.	.	.	.	C	.	.	.	T	.	.	.	.	A	-	.	C	.	C	.	.	.	C	T	.	.	.	.	.	.	G	.	T	.	.	.	-	C	.	C	A	.	.	.	G	.	.	.	.	.	.	.	.	.
OF54	.	.	i_6	A	.	.	T	.	.	.	.	.	.	A	A	G	.	.	.	C	.	.	.	.	$(A)_8$	.	.	.	-	-	A	.	.	A	T	.	.	.	.	T	.	.	.	T	C	.	.	A	.	.	.	.	C	.	.	.	.	.	.	.	.	A	A	.	C	.	C	.	.	T	C	T	.	T	.	.	.	.	.	.	.	C	.	.	-	.	.	C	.	.	.	.	.	.	.	.	.	.	.	.	.	.
OF58	.	.	i_6	.	C	.	T	.	.	.	.	.	.	.	A	.	.	.	.	C	C	C	.	.	$(A)_5$	.	.	.	.	.	.	.	.	.	.	.	.	.	.	T	C	C	.	.	.	G	T	.	.	.	T	.	C	.	.	.	.	T	.	.	.	A	-	.	C	.	C	.	.	.	C	T	.	T	.	.	.	T	.	.	.	C	.	.	-	.	.	C	.	.	.	.	.	.	.	.	G	.	.	.	.	T
OF60	.	.	i_6	.	.	.	T	.	T	.	.	.	.	A	A	.	.	.	.	C	.	.	.	A	$(A)_7$	.	.	A	.	.	.	.	.	.	.	.	.	.	.	T	.	.	.	.	.	.	.	.	.	.	.	.	C	.	.	.	.	.	.	.	.	A	-	.	C	C	.	.	.	.	C	.	.	.	.	.	T	.	.	.	.	.	.	.	-	.	.	C	.	.	.	.	.	.	.	.	G	.	G	T	.	.
OF78	C	.	i_6	.	.	.	T	A	.	.	.	.	.	A	A	.	.	.	.	C	.	C	.	A	$(A)_7$	.	.	.	.	.	.	.	.	.	.	.	C	.	.	T	C	C	.	.	.	.	.	.	.	.	.	.	C	T	.	.	.	.	.	.	.	A	-	.	C	.	C	.	.	.	C	.	.	.	.	.	T	.	.	.	.	C	.	.	-	.	.	C	.	.	.	T	.	.	.	.	.	.	.	.	.	.
Dmad	.	.	-	.	.	.	T	.	.	.	.	.	.	A	A	.	.	.	.	C	.	.	.	A	$(A)_7$	.	T	.	.	.	.	.	.	.	T	.	.	.	.	T	C	C	.	.	.	G	.	.	.	.	.	.	.	.	.	.	.	T	.	.	.	A	A	.	C	.	C	.	.	.	C	.	.	.	.	.	.	.	.	.	.	-	-	.	-	.	.	C	.	.	.	.	.	.	.	.	.	.	.	.	.	.
Dgua	.	.	-	.	.	.	T	.	.	.	.	.	.	A	A	.	.	-	.	C	.	.	.	.	$(A)_7$	.	T	.	.	.	G	.	.	.	T	.	.	T	.	T	C	C	.	.	A	G	.	.	.	.	.	.	.	.	.	.	.	T	.	A	.	A	A	.	C	.	C	.	.	.	C	.	.	.	.	.	.	.	.	.	.	.	.	.	-	.	.	C	.	.	.	.	.	.	.	.	G	.	.	.	.	.

Figure S4. *Caf1-55* and *Caf1-55dup* nucleotide polymorphic sites in 14 and 16 lines of *D. subobscura*, respectively. The first column indicates the name of each sequenced *D. subobscura* line. Numbers show nucleotide sites along the multiple alignment. Dots indicate the same nucleotide as in the OF01 sequence used as reference and dashes alignment gaps. Polymorphic sites marked with an asterisk correspond to nonsynonymous polymorphisms and the site marked with a cross in *Caf1-55dup* represents the gain of a stop codon. Information for the polymorphic sites in the *D. madeirensis* and *D. guanche* sequences used as outgroups is also shown shadowed in grey. i_n = insertion of n nucleotides, d_n = deletion of n nucleotides, $(A)_n$ = n repeats of adenine, ? = not sequenced. Bars above both tables indicate if a certain site belongs to 5'=5' flanking region; I1= first intron; E2= second exon; I2= second intron; E3= third exon or 3'= 3' flanking region.

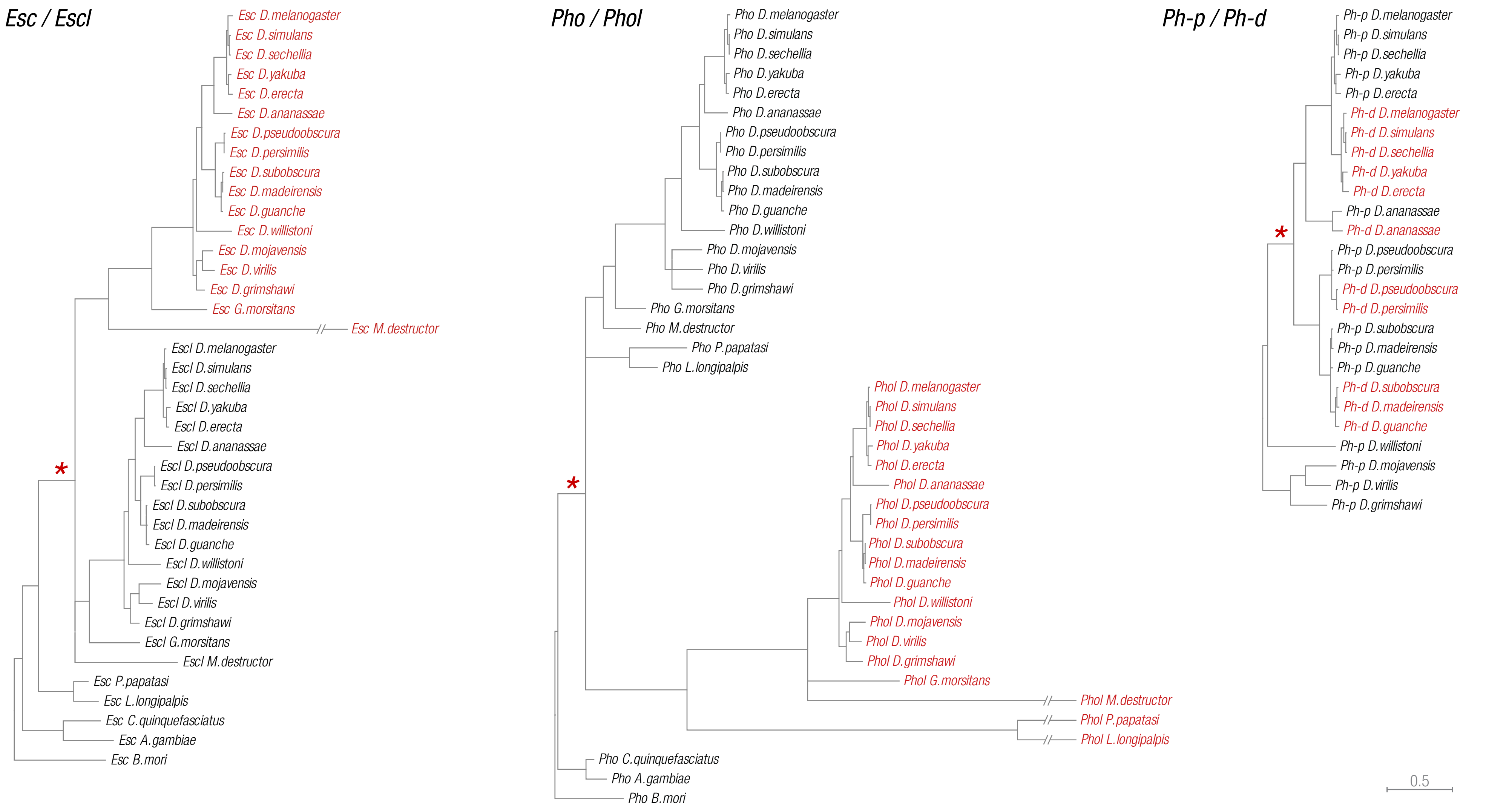

Figure S5. Phylogenetic trees according to nucleotide divergence of the *Esc*/*Escl*, *Pho*/*Phol* and *Ph-p*/*Ph-d* paralogs. Branch lengths were obtained with MEGA6 by maximun likelihood fixing the commonly accepted phylogeny of the studied species. The ancestral genes are represented in black and the derived duplicates in red. The scale at the lower right corner of the figure that indicates nucleotide substitutions per site is common for the three trees. Asterisks indicate the nodes where the duplication events occurred. Branches too long relative to the other branches are interrupted by a double dash.

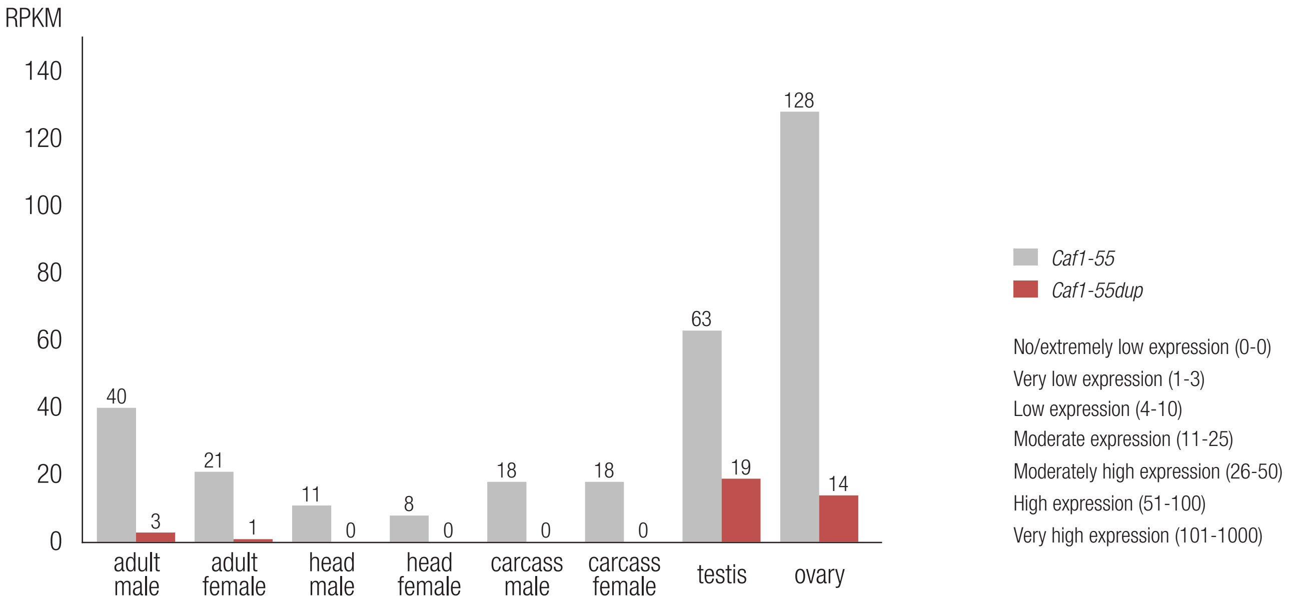

Figure S6. Expression levels of *Caf1-55* and *Caf1-55dup* in *D. pseudoobscura,* according to the RNA-Seq data available in FlyBase. Values correspond to reads per kilobase of exon per million mapped reads (RPKM). The experiments for each tissue are independent. Thus, RPKM values for the whole adult flies do not correspond to the sum of the RPKM values for the separate tissues. Testis or ovary were not included when carcass expression was analyzed. Classification of the expression levels, from “no expression” to “very high expression”, is given according to FlyBase.

Table S1. Primers used to amplify and sequence the studied genes, as well as PCR conditions.

Caf1-55

	Amplification primers	Annealing temperature [a]	Extension time [b]	Sequencing primers
D. subobscura	C1_F1 5' CAGCCGAGATTCAGTCAGC 3'	56 ºC	1'20"	C1_F1 5' CAGCCGAGATTCAGTCAGC 3'
D. madeirensis	C1_R1 5' CCATCTTCGGCATCCTCTG 3'			C1_R1 5' CCATCTTCGGCATCCTCTG 3'
D. guanche				C1_F2 5' CGGTGAATGCCAGCCAGAT 3'
	C1_F2 5' CGGTGAATGCCAGCCAGAT 3'	56 ºC	1'20"	C1_R2 5' TCAGCACATCGCCCTATCA 3'
	C1_R2 5' TCAGCACATCGCCCTATCA 3'			C1_SR1 5' CCTTCTCGTTGTCATAGTG 3'
				C1_SR2 5' CACAAGCAGATGGTATGAT 3'
				C1_SR3 5' GGAAGAGGCAAGAATGGTC 3'
				C1_SR4 5' CCAGAGCGTGTGTCATTAC 3'
				C1_SR5 5' TGCTGCTGCCCAAAGTATG 3'

GL12106

	Amplification primers	Annealing temperature [a]	Extension time [b]	Sequencing primers
D. persimilis	C1_F2 5' CGGTGAATGCCAGCCAGAT 3'	54 ºC	2'00"	C1_F2 5' CGGTGAATGCCAGCCAGAT 3'
	C1_R3 5' CCACTGCCAGGATCGTTCT 3'			C1_R3 5' CCACTGCCAGGATCGTTCT 3'
				C1_R2 5' TCAGCACATCGCCCTATCA 3'
				C1_SR3 5' GGAAGAGGCAAGAATGGTC 3'

Caf1-55dup

	Amplification primers	Annealing temperature [a]	Extension time [b]	Sequencing primers
D. subobscura	C1D_F1 5' GTTTCCGCTTGGCATGTCATAC 3'	54 ºC	2'10"	C1D_F1 5' GTTTCCGCTTGGCATGTCATAC 3'
D. madeirensis	C1D_R1 5' ACACATCTTGACCGTTGACA 3'			C1D_R1 5' ACACATCTTGACCGTTGACA 3'
D. guanche				C1D_SF1 5' CTGTACGATGAGATTGTGAC 3'
				C1D_SR1 5' TCGGACACGGAGCACATGAT 3'
				C1D_SF2 5' ATTGCCTTGTGGGATATG 3'
				C1D_SR2 5' AGCGTTGTGTCCACTGAA 3'
				C1D_SR3 5' ACACCAGGACATCGCTCTT 3'
				C1D_SR4 5' AGCCACTGGACCGTAAAGGAT 3'
D. miranda	C1D_F2 5' AGGGAGCGAAATGTGCAATC 3'	54 ºC	40"	C1D_F2 5' AGGGAGCGAAATGTGCAATC 3'
	C1D_R2 5' ATGTAGCGAGCACGGTTCAC 3'			C1D_R2 5' ATGTAGCGAGCACGGTTCAC 3'

Caf1-55dup cDNA

	Amplification primers	Annealing temperature [a]	Extension time [b]	Sequencing primers
D. pseudoobscura	C1D_F3 5' GTCATCATCAACCACGAAGG 3'	57 ºC	40"	C1D_F3 5' GTCATCATCAACCACGAAGG 3'
D. persimilis	C1D_R3a * 5' CATTTGCCACACTTCCATCA 3'			C1D_R3a 5' CATTTGCCACACTTCCATCA 3'
				C1D_SR5a 5' TATCCCACAAGGCGACAGT 3'
D. subobscura	C1D_F3 5' GTCATCATCAACCACGAAGG 3'	57 ºC	40"	C1D_F3 5' GTCATCATCAACCACGAAGG 3'
D. madeirensis	C1D_R3b * 5' TCCATGAGAAGTCGTTGATT 3'			C1D_R3b 5' TCCATGAGAAGTCGTTGATT 3'
D. guanche				C1D_SR5b 5' ATCCCACAAGGCAATAGTC 3'

* Used to synthesize the cDNA

Common PCR conditions

	Initial denaturation	32 cycles			Final extension
		Denaturation	Annealing	Extension	
	94 ºC	96 ºC	a	65 ºC	65 ºC
	5'00"	10"	10"	b	5'00"
